# LRLSHMDA: Laplacian Regularized Least Squares for Human Microbe–Disease Association prediction

**DOI:** 10.1038/s41598-017-08127-2

**Published:** 2017-08-08

**Authors:** Fan Wang, Zhi-An Huang, Xing Chen, Zexuan Zhu, Zhenkun Wen, Jiyun Zhao, Gui-Ying Yan

**Affiliations:** 1 School of Mechatronic Engineering, China University of Mining and Technology, Xuzhou, 221116 China; 2 Jiangsu Key Laboratory of Mine Mechanical and Electrical Equipment, China University of Mining and Technology, Xuzhou, 221116 China; 3 College of Computer Science and Software Engineering, Shenzhen University, Shenzhen, 518060 China; 4 School of Information and Control Engineering, China University of Mining and Technology, Xuzhou, 221116 China; 5 Academy of Mathematics and Systems Science, Chinese Academy of Sciences, Beijing, 100190 China

## Abstract

An increasing number of evidences indicate microbes are implicated in human physiological mechanisms, including complicated disease pathology. Some microbes have been demonstrated to be associated with diverse important human diseases or disorders. Through investigating these disease-related microbes, we can obtain a better understanding of human disease mechanisms for advancing medical scientific progress in terms of disease diagnosis, treatment, prevention, prognosis and drug discovery. Based on the known microbe-disease association network, we developed a semi-supervised computational model of Laplacian Regularized Least Squares for Human Microbe–Disease Association (LRLSHMDA) by introducing Gaussian interaction profile kernel similarity calculation and Laplacian regularized least squares classifier. LRLSHMDA reached the reliable AUCs of 0.8909 and 0.7657 based on the global and local leave-one-out cross validations, respectively. In the framework of 5-fold cross validation, average AUC value of 0.8794 +/−0.0029 further demonstrated its promising prediction ability. In case studies, 9, 9 and 8 of top-10 predicted microbes have been manually certified to be associated with asthma, colorectal carcinoma and chronic obstructive pulmonary disease by published literature evidence. Our proposed model achieves better prediction performance relative to the previous model. We expect that LRLSHMDA could offer insights into identifying more promising human microbe-disease associations in the future.

## Introduction

A microbe or microorganism refers to a microscopic living organism which could be single-celled or multicellular. With the deepening of research, microbes were basically classified into several species: bacteria, fungi, viruses, archaea, protozoa and others^1, 2^. As we all know, microbes are ubiquitously living in every part of biosphere, such as soil, rock, hot springs, even “seven miles deep” in the ocean. Therefore, it is not surprising that multitudes of commensal microbes colonize in human body, such as skin^3^, lung^4^, gut^5^ and oral cavity^6^. It is generally believed that microbial cells in our body outnumber our own cells by a ratio of 10-to-1^7^. In fact, most of these microbes not only do no harm to human health but also have a mutualistic symbiotic relationship with their human hosts, so called “forgotten organ”^8^. With the advent of high-throughput sequencing technology and analytic system, people have realized the critical role of microbe and carried out related investigations. It has been found that human microbes participate in many biological processes including energy harvest and storage, immune structure and function, protection against invasion by alien microbes and some important metabolic functions like fermenting and absorbing undigested carbohydrates^9, 10^. Therefore, “sick” microbial communities tend to cause physiological disorders of the human body. In other words, there may exist a potential association relationship between the dysbiosis of microbial communities and the occurrence of complex human diseases.

Over millennia, since the mutualistic symbiotic relationship was naturally selected and developed by evolutionarily ancient symbiosis of human and their commensal microbiota, they have been mutually affected by diverse interactions in many aspects. The commensal microbial community in human body could be greatly affected by the genetics and living environments (e.g. diets^11–14^, antibiotics^15^, season^16^ and smoking^17^) of their human host. For example, food sources i.e. diets, are the most important determinant for shaping the composition of the human intestinal microbiota. Extreme short-term diets could rapidly lead to a remarkable alteration in the composition of human intestinal microbiota, especially when lacking of carbohydrates. In addition, a dynamic balance of human microbiota is essential to maintain a good physical condition, which means that once such dynamic balance is broken, related human diseases and disorders may be induced. Based on the development of sequencing technology and analytic system such as 16S ribosomal RNA (rRNA) gene sequence and taxonomic profiles^18, 19^, human microbes have been identified to be related to some important diseases such as central nervous system disorder^20^, kidney stones^21^, cardiovascular disease^22^, psoriasis^23^, cancer^24^ and metabolic syndrome (e.g. obesity^25^ and diabetes^26, 27^). For example, as we know, oral cavity is a perfect habitat for a wide variety of oral microbiome including pathogenic bacteria, whose proliferation may give rise to an inflammatory disease, i.e. periodontitis^28^. Researchers compared gene expression difference of both periodontitis-related diseased samples and healthy samples. The results demonstrated that periodontitis-related microbial communities have highly conserved changes in metabolic and virulence gene expression profiles, whereas healthy samples do not. It means that community composition changes in oral microbiome could be implicated in the pathogenesis of periodontitis^29^. Furthermore, the gut flora has been found to have association with the pathologic end stage of chronic liver disease, i.e. liver cirrhosis. Through real-time quantitative polymerase chain reaction (qPCR) and 454 pyrosequencing of 16S rRNA V3 region, experiments showed that the fecal microbial communities are distinct in the cirrhosis-related samples, relative to the healthy samples^30^. Some pathogenic bacteria, such as *Proteobacteria* and *Fusobacteria*, are highly enriched in the cirrhosis patients potentially affecting their prognosis. It was reported that the predominant acquisition of *Helicobacter pylori* in the childhood could reduce the risk of allergy. The colonization with *Helicobacter pylori* was demonstrated to have an inverse association with the symptom of allergy, such as sensitization to pollens and molds^31^. Besides, metastasis is considered as the major reason of mortality from cancer. Because of Genome sequencing and computational analysis, it is feasible and helpful to conduct computational dissection of clones from tumors^32, 33^. Most importantly, a tumor’s metabolically compromised microenvironment is served as a haven harboring plenty of anaerobic bacteria, which localize and cause lysis in transplanted tumors. Combination bacteriolytic therapy (called COBALT) is regarded as a new weapon against cancer when systematically administered with conventional drugs and chemotherapeutics^34^. Therefore, it is anticipated that using bacteria could help control the formation of fast-growing clones, although there are some potential problems with COBALT need to be fixed, such as: toxicity and drug resistance.

Considering the medical value of disease-related microbiota, some large-scale sequencing projects, such as the Human Microbiome Project (HMP)^35^ and the Earth Microbiome Project (EMP)^36^, attempted to investigate the relationship between microbiota and human diseases. Launched in 2008, HMP was aimed at identifying and characterizing those microorganisms which have a strong association with human health and disease. Reference genomic sequencing of 3000 individual bacterial isolates was identified for further metagenomic comparison analysis. Moreover, some related databases^37–40^ have been developed to collect and manage the biological information about disease-related microbes. A human microbe–disease association database called HMDAD^41^ manually integrated 483 disease-microbe association entries at the genus level based on previously published literatures. These databases are regarded as the essential tools for capturing and analyzing the rapidly accumulating information for microorganisms, which provides a possibility for large-scale disease-related prediction. However, the identification of the known microbe-disease associations is only a tip of the iceberg which indicates that little effort has been done to fully understand the pathology of diverse human diseases from a microbial perspective. It may slow down the development of disease diagnosis, treatment, prevention, prognosis and drug discovery. Currently, culture-independent approaches and quantitative methods are widely used in the characterization of microbial community. However, only depending on these conventional methods is not only laborious but also time-consuming. It is difficult to fully explore the potential microbe-disease associations in a short term. For predicting most probable associations, computational approach, served as an assistant tool, has achieved remarkable results in other biological domains, such as drug-target interaction prediction^42, 43^, synergistic drug combination inference^44^, non-coding RNA (ncRNA)-disease association prediction^45–48^, gene-disease association prediction^49^, protein-protein interaction prediction^50^, ncRNA-environmental factor interaction prediction^51^. Recently, increasing attention has been paid to computational biology for microbe-disease association^52–56^. These computational methods have been developed to facilitate relevant research in different ways, such as: the package for implementing community-level metabolic network reconstruction, the computational methodology for predicting the influence of microbial proteins in human biological events, the computational framework for identification of key functional differences in microbiome-related disease, the web application for annotation and analysis of specific genes in the human gut microbiome. In 2016, we have presented the first computational model called KATZHMDA^57^ in this domain based on KATZ method, which specializes in social network prediction^58^. Based on the heterogeneous graph constructed by known microbe-disease association network, microbe similarity network and disease similarity network, we integrated the number of walks and their own lengths regarded as an effective measure index for calculating the potential association probability between microbes and diseases. Its reliable prediction performance makes us believe that computational approach could effectively contribute to inferring potential microbe-disease associations.

**Figure 1:**
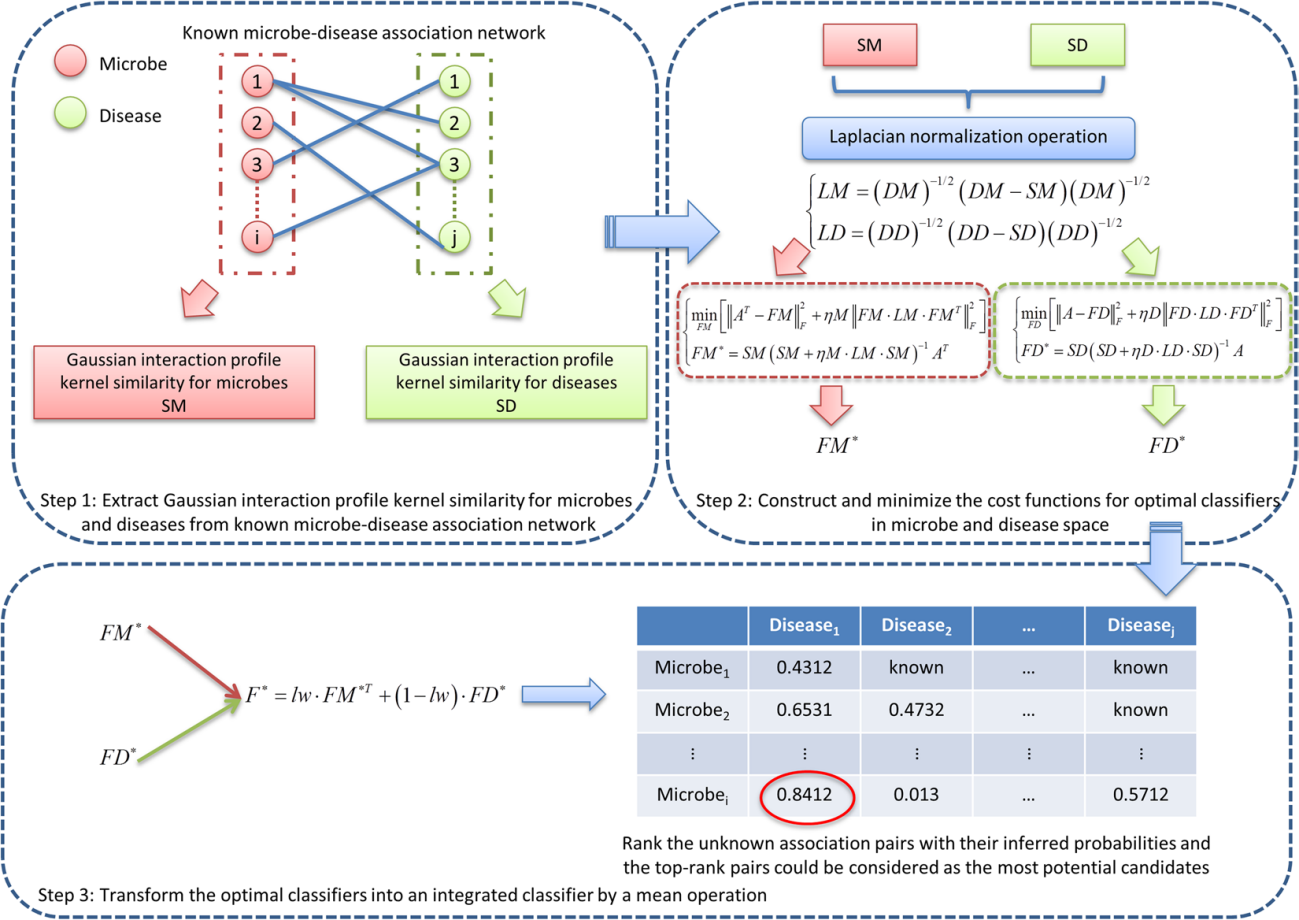
Flowchart of LRLSHMDA. Based on the known microbe-disease association network, we utilized Gaussian interaction profile kernel similarity and LapRLS classification to infer the potential microbe-disease associations.

In this article, we aimed to utilize the computational prediction model for inferring the most potential microbe-disease associations by prioritizing their association probability values based on the known microbe-disease association network. These promising microbe-disease associations could be given priority for further experimental verification. It is anticipated that introducing computational prediction models could accelerate the identification of novel microbe-disease association. Therefore, we developed a novel computational model of Laplacian Regularized Least Squares for Human Microbe–Disease Association (LRLSHMDA) based on the known human microbe-disease association network derived from the HMDAD database (See Fig. 1). Because of the lack of negative samples i.e., those microbes are verified to have no association with a given disease, a semi-supervised learning framework is adopted in the proposed model. By introducing Gaussian interaction profile kernel similarity and Laplacian regularized least squares (LapRLS) classification, topology structures in the known microbe-disease association network can be utilized to effectively exploit the implicative information of vertices and edges, which helps train the optimal classifier. As a global measure approach, our model enables to simultaneously prioritize all candidate microbe-disease pairs for all investigated diseases. As a result, we conducted cross validations and case studies on the proposed model for evaluating the prediction performance. Promising validation results demonstrated that LRLSHMDA could be an effective tool to advance the identification of disease-related microbes and aid future research focus towards a mutualistic symbiotic relationship between microorganisms and their human host.

## Results

### Cross validation

To comprehensively evaluate the prediction performance, leave-one-out cross validation (LOOCV) and 5-fold cross validation (5-fold CV) were conducted on the proposed model. LOOCV was implemented on the known verified microbe-disease association pairs, each of which was left out in turns to be a test sample when others were used for training model. If the test sample is ranked higher than the specific threshold, it could be considered to make a correct prediction for this test microbe-disease association pair. Two types of LOOCV called global LOOCV and local LOOCV were conducted in this study based on the diverse ranking scopes. In terms of global LOOCV, the test sample was ranked among all unknown candidate microbe-disease association pairs involved in all investigated diseases. In terms of local LOOCV, the test sample was only ranked among other unknown disease-related microbes for a given disease. Namely, the major difference between two types of LOOCV is whether all investigated diseases are considered or not. In addition to LOOCV, 5-fold CV was also introduced to evaluate the performance of the proposed model. We randomly split up all known verified microbe-disease association pairs into five groups. Instead of selecting one microbe-disease association as a test sample, each of these five groups was selected in turns to be test samples while other four groups were served as the training samples. To reduce the bias caused by such random divisions, this process was conducted 100 times in the framework of 5-fold CV. For visually evaluating the performance, receiver-operating characteristics (ROC) curve, which is a common means for evaluating the binary classification models, was therefore adopted in our study. Sensitivity and specificity are two key measure indexes used in ROC curve. In this study, sensitivity measures the proportion of a test to correctly identify those microbe-disease associations, whereas specificity measures the proportion of a test to correctly identify those microbes without the known associations with the investigated diseases. In this way, we plotted ROC curve by using true positive rate (sensitivity) versus false positive rate (1-specificity) at gradually changing thresholds. The area under ROC curve (AUC) was also commonly calculated for measuring performance. Generally, AUC = 0.5 shows a purely random performance while AUC = 1 represents a completely perfect performance.

**Figure 2:**
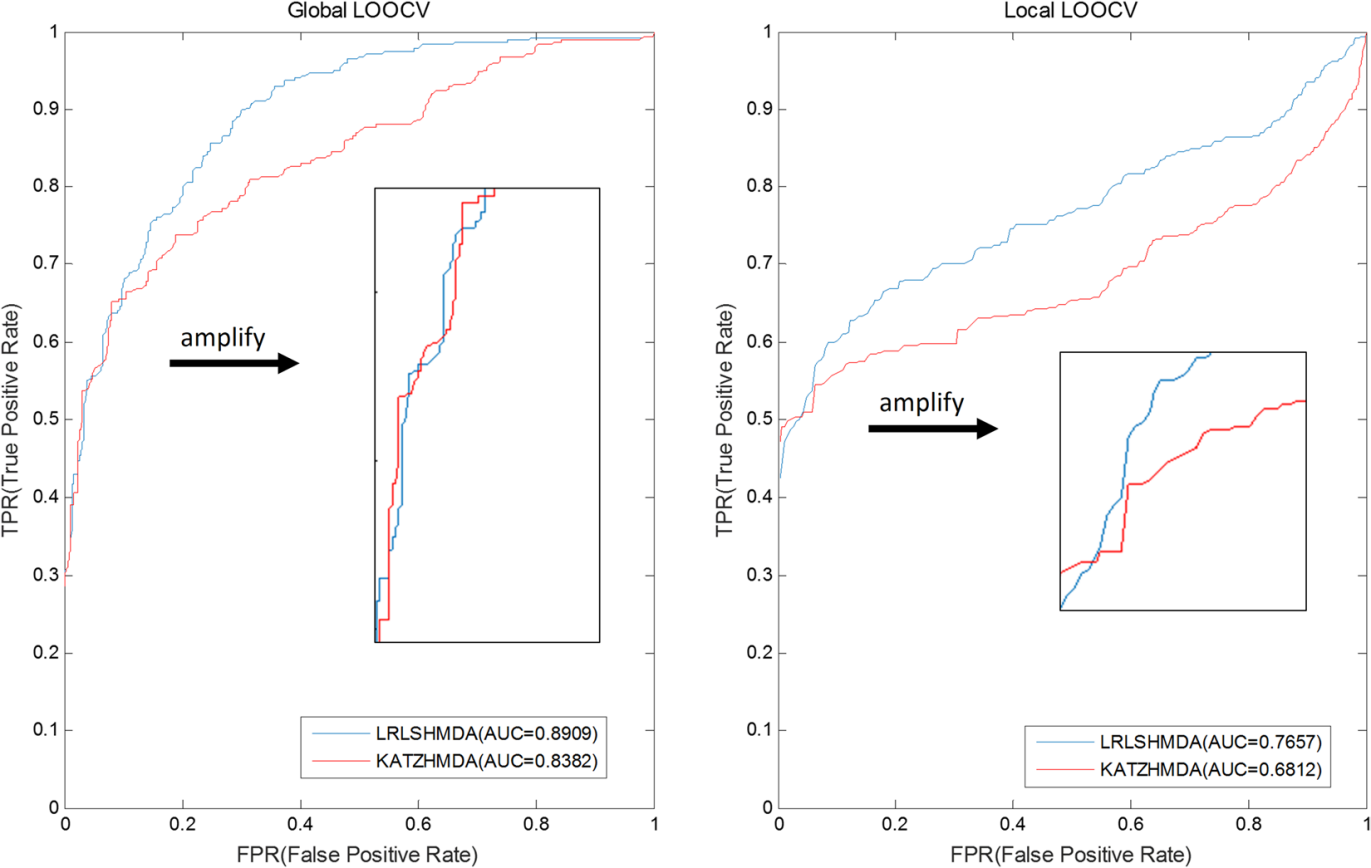
Prediction performance comparison between LRLSHMDA and KATZHMDA in the frameworks of both global and local LOOCV.

As we have seen in Fig. 2, AUCs of 0.8909 and 0.7657 in the proposed model demonstrated its reliable prediction performance based on global and local LOOCV, respectively. Compared with KATZHMDA’s result (AUCs of 0.8382 and 0.6812 in global and local LOOCV), our newly proposed model obtained a better improvement. In the framework of 5-fold CV, the average AUC of 0.8794 +/−0.0029 further shows the more reliable prediction performance, relative to KATZHMDA’s (the average AUC of 0.8301 +/−0.0033). This result reveals that, although these two approaches are both based on the bipartite graph, LRLSHMDA indeed performs better in terms of the prediction accuracy.

### Case studies

To measure the practical effect of LRLSHMDA, we selected three important human diseases in our case studies for revealing the pathological relationship between microbes and respiratory system as well as digestive system from a microbial perspective. As a result, 9, 9 and 8 of top-10 predicted microbes have been supported to be associated with the onset of asthma, colorectal carcinoma and chronic obstructive pulmonary disease (COPD) based on previously published literatures.

Asthma, a common chronic lung disease inducing the inflammation and narrowing the airways, resulted in 489,000 global deaths in 2013^59^. Asthma often starts during childhood with multiple symptoms such as shortness of breath, recurring periods of wheezing, chest tightness and coughing at night or early in the morning. In recent years, besides the well-known causes of genetic and environmental factors^60^, the involvement of microbe in pathology of asthma was demonstrated by increasingly emerging evidences^61, 62^. For example, the changing populations of *Sphingomonadaceae*, *Comamonadaceae*, *Oxalobacteraceae* and other bacterial families are potentially associated with bronchial hyperresponsiveness in patients with suboptimally controlled asthma. To evaluate the prediction effect on asthma, we implemented a case study of asthma based on our approach. In the prediction list, 9 of top-10 predicted microbes have been verified to have an impact on the asthmatic patients (see Table 1). For example, chronic airway infection with *Pseudomonas aeruginosa* (1st in the prediction list) has some prophylactic effect on bronchial asthma^63^. The colonization of *Clostridium difficile* (2ed in the prediction list) in infants at age 1 month was found to have association with wheeze, eczema and asthma at age 6 to 7 years^64^. *Lactobacillus* (3rd in the prediction list) could inhibit airway inflammation in an ovalbumin (OVA)-induced murine model of asthma. This finding may offer an insight into the potential prevention action of asthma^65^. *Actinobacteria* (5th in the prediction list) and *Firmicutes* (6th in the prediction list) were found to have a lower proportion in asthmatic patients, relative to non-asthmatic people^66^.

**Table 1:** In the case study of asthma, 9 out of top-10 predicted microbes have been supported by literature evidences.

Rank	Microbe	Evidence
1	Pseudomonas	PMID:9294308
2	Clostridium difficile	PMID:21872915
3	Lactobacillus	PMID:20592920
4	Burkholderia	unconfirmed
5	Actinobacteria	PMID:23265859
6	Firmicutes	PMID:23265859
7	Clostridium coccoides	PMID:21477358
8	Clostridia	PMID:21477358
9	Staphylococcus aureus	PMID:17950502
10	Bifidobacterium	PMID:24735374

Known as bowel cancer, colorectal carcinoma starts in the colon or rectum, which belongs to the parts of the large intestine. Patients with colorectal carcinoma may appear following symptoms, such as blood in the stool, loss of appetite and weight, worsening constipation, nausea and emesis^67^. Multiple risk factors could induce colorectal carcinoma such as smoking, diet, obesity, lack of physical activity and alcohol. Colorectal carcinoma reaches nearly 65 percentages of five-year survival rates and becomes the third most common type of cancer in United States. In 2012, it caused 1.4 million new cases and 694,000 deaths all over the world^68^. Some evidences revealed that microbes play an increasingly significant role in the onset of colorectal carcinoma. For example, lecithinase-negative *Clostridium* and *Lactobacillus* were identified to be more abundant in colorectal carcinoma patients^69^. Some *Lactobacillus* species and *Eubacterium aerofaciens* have an effect to reduce the disease risk^70^. To examine the adverse and beneficial microbes in digestive system, a case study of colorectal carcinoma was conducted on our model. As a result, 9 of top-10 predicted microbes have been proven based on the experimentally verified evidences (see Table 2). For example, infection with *Helicobacter pylori* (2ed in the prediction list) could increase the risk of colorectal carcinoma^71^. The dramatic increase of *Clostridium difficile* (3th in the prediction list) and *C*. *coccoides* (4th in the prediction list) was a potential pathogenic factor for triggering colorectal carcinoma^72^. *Staphylococcus aureus* (5th in the prediction list) as the immunoadsorbent can be applied to therapy for a patient with a metastatic colon carcinoma^73^. *Bifidobacterium* (6th in the prediction list) may protect against the development of colon carcinoma^74^.

**Table 2:** In the case study of colorectal carcinoma, 9 out of top-10 predicted microbes have been supported by literature evidences.

Rank	Microbe	Evidence
1	Proteobacteria	unconfirmed
2	Helicobacter pylori	PMID:11774957
3	Clostridium difficile	PMID:19807912
4	Clostridium coccoides	PMID:19807912
5	Staphylococcus aureus	PMID:7074582
6	Bifidobacterium	PMID:9111222
7	Haemophilus	PMID:22761885
8	Actinobacteria	PMID:24316595
9	Lactobacillus	PMID:15828052
10	Veillonella	PMID:22761885

COPD, a type of obstructive lung disease, progressively makes it hard to breathe^75^. Besides most COPD patients smoke or used to smoke, air pollution, cooking fires and genetics could also result in narrowing of the small airways and breakdown of lung tissue, which can bring on typical symptoms including cough with sputum production and shortness of breath. In 2013, COPD was the third leading cause of death all over the world, causing 2.9 million deaths. Especially, more than 90% of deaths occur in the developing countries. Most importantly, 329 million people (about 5% of global population) were hindered by COPD in the world^59, 76^. However, there is still no known cure and pathogenesis for this disease. Recent discoveries^77, 78^ suggested that shifts or perturbations in the microbiota may play an important role in the development of COPD. For example, two types of bacterial microbiota *Proteobacteria* and *Firmicutes* were speculated to be related to COPD. Currently, little is known about the COPD-related microbes, which motivated us to conduct a case study of COPD on the proposed model. Eight of top-10 predicted microbes in the prediction list obtained evidence support (see Table 3). For example, some experiment results showed that *Helicobacter pylori* (1st in the prediction list), *Clostridium difficile* (2ed in the prediction list) and *Comamonadaceae* (8th in the prediction list) may be implicated in COPD^77, 79, 80^. The significant expansion in *Actinobacteria* (4th in the prediction list), *Staphylococcus* (5th in the prediction list), *Firmicutes* (6th in the prediction list) and *Sphingomonadaceae* (10th in the prediction list) was proven to drive the development of COPD^81–83^. Furthermore, a decrease in *Clostridia* (7th in the prediction list) could result in the exacerbation of COPD.

**Table 3:** In the case study of COPD, 8 out of top-10 predicted microbes have been supported by literature evidences.

Rank	Microbe	Evidence
1	Helicobacter pylori	PMID:15733502
2	Clostridium difficile	PMID:15655746
3	Clostridium coccoides	unconfirmed
4	Actinobacteria	PMID:26852737
5	Staphylococcus	PMID:15338798
6	Firmicutes	PMID:24591822
7	Clostridia	PMID:26852737
8	Comamonadaceae	PMID:20141328
9	Oxalobacteraceae	unconfirmed
10	Sphingomonadaceae	PMID:26852737

We also compared the performance between LRLSHMDA and KATZHMDA for the case studies of these three diseases by manually verifying the top-10 disease-related microbes inferred by KATZHMDA based on the literature evidences as well (see Supplementary Table 4). Based on KATZHMDA model, 4, 5 and 5 of top-10 predicted microbes have been supported to be linked with the onset of these three diseases. The comparative result of the top-10 prediction list was shown in Table 4. According to this comparative result, we believe that LRLSHMDA indeed possesses a relatively higher accuracy rate for inferring potential disease-related microbes.

**Table 4:** Performance comparison between LRLSHMDA and KATZHMDA in the case studies of the confirmation of the top-10 prediction list for three human complex diseases.

Model	Asthma	Colorectal carcinoma	COPD
LRLSHMDA	9	9	8
KATZHMDA	4	5	5

Above all, we conclude that human microbes participate in the regulation of multiple host physiological activities. Once the dynamic change of microbial communities is disturbed by other environmental factors and therefore becomes unbalanced, it could lead to human related diseases or disorders. Understanding how microbes affect their human hosts could shed a light on complicated diseases. Besides these three case studies, LRLSHMDA as a global measure model can simultaneously prioritize the potential microbes related to other investigated diseases, such as diabetes, liver cirrhosis, periodontal, obesity and eczema. These published prediction results were ranked based on their association probabilities (see Supplementary Table 5). We hope that it can provide researchers an aid to guide the experimental verification for further accelerating the detection of potential disease-related microbes.

## Discussions

Human body is a home that harbors thousands of microbe species constructing complicated microbial ecosystems, which have been known to affect human health. With the development of high-throughput sequencing technology and analytic system, researchers could carry out the further study (e.g. large-scale sequencing projects, disease-related microbe databases) for investigating the pathological relationship between microbes and their human hosts. However, what we have learned is a drop in the bucket, and that it is insufficient for us to thoroughly understand their pathogenic mechanism. Only depending on the culture-independent approaches and quantitative methods or other conventional experimental validation methods, is not only time-consuming but also laborious. As an effective tool, computational model has a great effect on the progress of many other biological domains. In this article, we proposed a novel semi-supervised learning computational model based on the framework of LapRLS. Gaussian interaction profile kernel similarity was adopted to extract the microbe similarity network and disease similarity network from the experimentally verified microbe-disease association network. By constructing and optimizing the cost functions in microbe space and disease space, the optimal classifier functions can be integrated to calculate the probability matrix, representing candidate microbe-disease association pairs with their predicted correlation coefficients. As a result, the proposed model achieved a reliable prediction performance in the evaluation frameworks of global LOOCV (AUC of 0.8909), local LOOCV (AUC of 0.7657) and 5-fold CV (average AUC value of 0.8794 +/−0.0029). In our case studies, 9, 9 and 8 of top-10 inferred microbes have been confirmed to have associations with asthma, colorectal carcinoma and COPD according to the literature evidence. As a global measure model, our model can simultaneously prioritize all candidate microbe-disease pairs. Given the promising prediction performance, we believe that LRLSHMDA could be regarded as an effective tool advancing the progress of biomedical identification of potential disease-related microbes. In the future, if the negative microbe-disease association data is available, the prediction performance could be further improved by adding negative values in microbe similarity and disease similarity for representing the adverse associations between themselves.

The reliable performance of our approach could well benefit from several major factors as follows. (1) We used Gaussian kernel interaction profiles to extract the potential similarity for microbes and diseases by making use of topology structures in known microbe-disease association network. (2) Smoothening the classifiers in microbe space and disease space, is a reasonable trade-off between bias and variance for obtaining the strong capability of fitting and generalization. (3) Based on the LapRLS framework, the proposed model is a semi-supervised learning method, i.e. the training data is regarded as labeled samples while other test data as unlabeled samples. By utilizing the known microbe-disease association pairs as labeled sample, it is feasible to adopt a semi-supervised learning algorithm, especially when negative microbe-disease association data is extremely scarce. (4) It is reasonable to integrate two separate optimal classifiers into a unified space by mean operation for improving the accuracy of the prediction.

Of course, there are some limitations inhibiting the performance of LRLSHMDA. (1) The experimentally verified microbe-disease association pairs used in our approach are relatively insufficient, so the sparse association network could affect the predictive capability. It is anticipated that this problem will be eased when collecting more microbe-disease associations in the future. (2) Although microbe similarity network and disease similarity network can be calculated by Gaussian interaction profile kernel similarity, it is difficult to avoid bias brought by such an inference. Given other substantial datasets such as: disease semantic similarity and microbe homologous sequence similarity, Gaussian interaction profile kernel similarity for microbes and diseases can be replaced to enhance the reliability of information resource. (3) The combination operation of those two optimal classifiers could be improved based on other more effective machine learning algorithms. (4) The proposed model cannot be applied to those microbes without any known related disease. (5) Only 39 diseases have been considered in the HMDAD database, which means that some microbes may have no relationship with all these 39 diseases but our model still make a prediction and prioritize these associations in the top rank. It may bring some misreadings that these microbes seem to be strongly associated with several of these 39 diseases but in fact they do not. This problem could be solved if more diseases are included. (6) Some microbes have been confirmed to play an important role in development, diagnosis, prevention, prognosis, and treatment for cancer^84–87^. Therefore, it is essential to investigate the effect of microbe on cancer. However, because there are few cancer-related entries in the HMDAD database, we cannot further explore the relationship between microbe and cancer at present.

## Methods

### Microbe-disease associations

By manually collecting microbe-disease association data set from previously published literatures, Ma *et al*. constructed the Human Microbe-Disease Association Database called HMDAD (http://www.cuilab.cn/hmdad)^41^ publicly providing 483 microbe-disease entries, which involve 39 diseases and 292 microbes (see Supplementary Table 1). 16S RNA sequencing was commonly used in human-associated microbiome studies, whose articles generally described related information at the genus level, so therefore most microbe names in HMDAD were recorded in genus as well. Based on these known microbe-disease entries, we defined an adjacency matrix as variable *Y* for representing their association relationship, i.e. *Y*(*i*,*j*) = 1 means microbe *i* is associated with disease *j*, and vice versa. For better description, two variables *nm* and *nd* are respectively defined as the numbers of microbes and diseases investigated in our study.

### Gaussian interaction profile kernel similarity for microbes

Considering any two microbes related with more common human diseases could tend to potentially share higher functional similarity, we used Gaussian kernel interaction profiles to calculate the inferred microbe similarity based on the topologic information of known microbe-disease association network. The interaction profiles of microbe *m*_*i*_ denoted as *IP*(*m*_*i*_) record the relationship between *m*_*i*_ and the all investigated diseases, i.e. the ith row of matrix *Y*. For two arbitrary microbes *m*_*i*_ and *m*_*j*_, their inferred similarity can be calculated based on their interaction profiles *IP*(*m*_*i*_) and *IP*(*m*_*j*_) as follows:

1 $${\rm{KM}}({m}_{i},{m}_{j})=\exp (-\,{\gamma }_{m}{\Vert IP({m}_{i})-IP({m}_{j})\Vert }^{2})$$

where parameter *γ*_*m*_ is responsible for controlling the kernel bandwidth. This parameter *γ*_*m*_ needs to be updated with the normalization operation of a novel bandwidth parameter *γ*′_*m*_ by the mean number of aggregate associations with diseases for each microbe:

2 $${\gamma }_{m}={\gamma }_{m}^{^{\prime} }/(\frac{1}{nm}\sum _{i=1}^{nm}\Vert IP({m}_{i}){\Vert }^{2})$$

Here, for simplified calculation, *γ*′_*m*_ was assigned to 1 according to the previous study^88^. In this way, *KM* matrix could be calculated to represent the inferred microbe similarity, i.e. *KM*(*i*,*j*) denotes how microbe *m*_*i*_ is potentially similar with microbe *m*_*j*_.

### Gaussian interaction profile kernel similarity for diseases

Similar to microbes, Gaussian interaction profile kernel similarity for diseases *KD* can be inferred as follows:

3 $${\rm{KD}}({d}_{i},{d}_{j})=\exp (-\,{\gamma }_{d}{\Vert IP({d}_{i})-IP({d}_{j})\Vert }^{2})$$

4 $${\gamma }_{d}=\gamma {^{\prime} }_{d}/(\frac{1}{nd}\sum _{i=1}^{nd}{\Vert IP({d}_{i})\Vert }^{2})$$

where *γ*′_*d*_ was also set to 1.

In particular, when implementing cross validation for performance evaluation, Gaussian interaction profile kernel similarity for diseases and microbes needs to be recomputed for those left-out known microbe-disease association pairs.

### LRLSHMDA

LapRLS framework is commonly applied in machine learning for minimizing the prediction error. We therefore developed a novel semi-supervised computational model of LRLSHMDA to prioritize the most potential microbe-disease associations. The proposed model followed the basic process, which was depicted in Fig. 1. Firstly, Gaussian interaction profile kernel similarity for microbes and diseases (i.e. *SM* and *SD*, see Supplementary Tables 2–3, one important fact should be pointed out here is that Gaussian interaction profile kernel similarity should be recalculated for each run in LOOCV) could be calculated based on the known microbe-disease association network originated from the HMDAD database. Secondly, *SM* and *SD* need to be normalized by the Laplacian operation. Thirdly, to make a better trade-off between bias and variance, we constructed the cost function, which was minimized to obtain the optimal classifiers in microbe space and disease space. Finally, these two optimal classifiers need to be transformed into an integrated classifier in unified space for calculating the probability matrix, which reflects the association potential of unknown microbe-disease pairs. Based on the inferred association probabilities, those microbe-disease pairs in the top rank could be considered as the most potential candidates. To obtain the strong capability of fitting and generalization, the classifier should be smooth in microbe space and disease space. Namely, between similar microbes/diseases and the same disease/microbe, the scores for these potential associations should be similar.

After the calculation of Gaussian interaction profile kernel similarity for microbes and diseases *SM* and *SD*, Laplacian operation was firstly used to normalize *SM* and *SD* as follows:

5 $${\rm{L}}{\rm{M}}={(DM)}^{-1/2}({\rm{D}}{\rm{M}}-{\rm{S}}{\rm{M}}){(DM)}^{-1/2}$$

6 $${\rm{L}}{\rm{D}}={(DD)}^{-1/2}({\rm{D}}{\rm{D}}-{\rm{S}}{\rm{D}}){(DD)}^{-1/2}$$

where *DM* and *DD* are diagonal matrices, whose entities *DM*(*i*,*i*) and *DD*(*j*,*j*) are the aggregates of the ith row of *DM* and jth row of *DD*, respectively.

Later, we defined cost functions in microbe space and disease space, which were depicted by the following formulas (7) and (8).

7 $${{\rm{\min }}}_{FM}[{\Vert {A}^{T}-FM\Vert }_{F}^{2}+\eta M{\Vert FM\cdot LM\cdot F{M}^{T}\Vert }_{F}^{2}]$$

8 $${{\rm{\min }}}_{FD}[{\Vert A-FD\Vert }_{F}^{2}+\eta D{\Vert FD\cdot LD\cdot F{D}^{T}\Vert }_{F}^{2}]$$

where ${\Vert \cdot \Vert }_{F}$ denotes the Frobenius norm and that *ηM* and *ηD* are the trade-off parameters, which were assigned to 1 based on the previously published literature^88^. As we have seen, formulas (7) and (8) described a minimum optimization problem, which could be solved by turning into a following optimal classification functions:

9 $$F{M}^{\ast }=SM{(SM+\eta M\cdot LM\cdot SM)}^{-1}{A}^{T}$$

10 $$F{D}^{\ast }=SD{(SD+\eta D\cdot LD\cdot SD)}^{-1}A$$

Finally, the optimal classifiers FM* and FD* were transformed into an integrated classifier in unified space with a simple weighted average operation as follows:

11 $${F}^{\ast }=lw\cdot F{M}^{\ast T}+(1-{\rm{lw}})\cdot F{D}^{\ast }$$

Here, parameter *lw* indicated that different weights were applied to the classification functions in microbe space and disease space. F* was a probability matrix (nm*nd) representing the predicted microbe-disease association network.

## Electronic supplementary material

Supplementary material

Supplementary Table 1

Supplementary Table 2

Supplementary Table 3

Supplementary Table 4

Supplementary Table 5
