# Supplementary material for: LRLSHMDA: Laplacian Regularized Least Squares for Human Microbe–Disease Association prediction
Source: Sci Rep. 2017 Aug 8;7:7601. doi: 10.1038/s41598-017-08127-2 (PMC5548838; doi:10.1038/s41598-017-08127-2)
Supplement: Supplementary file 1 — Supplementary material [file 41598_2017_8127_MOESM1_ESM.docx]

**LRLSHMDA: Laplacian Regularized Least Squares for Human Microbe–Disease Association prediction**

Fan Wang^1,2, #^, Zhi-An Huang^3, #^, Xing Chen^4, *^, Zexuan Zhu^3,*^, Zhenkun Wen^3^, Jiyun Zhao^1^, Gui-Ying Yan^5^

^1^ School of Mechatronic Engineering, China University of Mining and Technology, Xuzhou, 221116, China

^2^ Jiangsu Key Laboratory of Mine Mechanical and Electrical Equipment, China University of Mining and Technology, Xuzhou, 221116, China

^3^ College of Computer Science and Software Engineering, Shenzhen University, Shenzhen, 518060, China

^4^ School of Information and Control Engineering, China University of Mining and Technology, Xuzhou, 221116, China

^5^Academy of Mathematics and Systems Science, Chinese Academy of Sciences, Beijing, 100190, China

*Corresponding author

#The authors wish it to be known that, in their opinion, the first two authors should be regarded as joint First Authors.

**Email**: [xingchen@amss.ac.cn](mailto:xingchen@amss.ac.cn); [zhuzx@szu.edu.cn](mailto:zhuzx@szu.edu.cn)

**Supplementary Information**

**Supplementary Table 1.** Microbe names, disease names and known microbe-disease associations derived from HMDAD database.

**Supplementary Table 2.** Gaussian interaction profile kernel similarity for microbes.

**Supplementary Table 3.** Gaussian interaction profile kernel similarity for diseases.

**Supplementary Table 4.** Based on KATZHMDA model, 4, 5 and 5 of top-10 predicted microbes have been supported to be linked with the onset of asthma, colorectal carcinoma and COPD based on previously published literatures.

**Supplementary Table 5.** As a global measure model, LRLSHMDA can simultaneously prioritize the potential disease-related microbes for all investigated diseases based on the association probabilities.
